# Synthesis, Structure and Electrical Resistivity of Carbon Nanotubes Synthesized over Group VIII Metallocenes

**DOI:** 10.3390/nano10112279

**Published:** 2020-11-17

**Authors:** Aida R. Karaeva, Sergey A. Urvanov, Nikita V. Kazennov, Eduard B. Mitberg, Vladimir Z. Mordkovich

**Affiliations:** Technological Institute for Superhard and Novel Carbon Materials, 7A Tsentralnaya Street, Troitsk, 108840 Moscow, Russia; karaevaar@tisnum.ru (A.R.K.); urvanov@tisnum.ru (S.A.U.); kazennov@tisnum.ru (N.V.K.); mitbergeb@tisnum.ru (E.B.M.)

**Keywords:** carbon, nanotube, metallocene, Fe, Co, Ni, cooperative effect, electrical resistivity

## Abstract

The paper reports the synthesis of carbon nanotubes from ethanol over group VIII (Fe, Co, Ni) catalysts derived from corresponding metallocenes. Several unexpected cooperative effects are reported, which are never observed in the case of individual metallocenes such as the commonly used ferrocene catalyst Fe(C_5_H_5_)_2_. The formation of very long (up to several µm) straight monocrystal metal kernels inside the carbon nanotubes was the most interesting effect. The use of trimetal catalysts (Fe_1-x-y_Co_x_Ni_y_)(C_5_H_5_)_2_ resulted in the sharp increase in the yield of carbon nanotubes. The electrical conductivity of the produced nanotubes is determined by the nature of the catalyst. The variation of individual metals in the Ni-Co-Fe leads to a drop of the electrical resistivity of nanotube samples by the order of magnitude, i.e., from 1.0 × 10^−3^ to 1.1 × 10^−5^ Ω∙m. A controlled change in the electrophysical properties of the nanotubes can make it possible to expand their use as fillers in composites, photothermal and tunable magnetic nanomaterials with pre-designed electrical conductivity and other electromagnetic properties.

## 1. Introduction

There is a great variety of carbon nanotube (CNT) synthesis techniques available in literature affording versatile morphology, sizes (diameter, length), unusual properties and structure. The most productive and cost-effective method is the catalytic chemical vapor deposition of nanotubes (CVD) [1,2]. The CVD method is based on the catalytic decomposition of carbon precursors and allows the synthesis of CNTs both on a substrate supported catalyst [3,4], and on a floating catalyst [5,6,7,8] aka aerosol synthesis. The choice of a catalyst for the synthesis of CNT plays an important role. The nature of the catalyst components, their preparation and the method of supply to the synthesis zone all affect the structural characteristics, morphology and properties of the product [9]. The growth of short CNTs is actively catalyzed by supported catalysts [10,11], while the growth of long CNTs is more usually provided by a floating catalyst [12].

Fe-, Co- and Ni-containing catalysts, or their combinations are most often used as active particles for the growth of nanotubes. Suitable precursors for floating catalysts are organometallic compounds, including the transition metal metallocenes Fe, Co and Ni. They are decomposed to form metal nanoparticles at relatively low temperatures. For the formation of nanotubes, the presence of such metal particles is especially important, since the inner diameter of a nanotube is determined by the size of the metal nanoparticle [13]. The advantage of using floating catalysts is also the decomposition of carbon precursors on the surface of active particles in the carrier gas flow and the formation of an aerogel consisting of vertically oriented arrays of long CNTs. The aerosol method in a continuous mode can synthesize carbon nanotubes in macro-volumes.

The aerosol synthesis is known as being capable of producing a “flexible smoke” of millimeter-long [14,15] or even centimeter-long [16,17] CNTs of relatively small diameter, usually dominated by single-walled or double-walled tubes. In these publications, the method is based on the decomposition of a carbon precursor and the nucleation of carbon nanotubes on Fe nanoparticles in a hydrogen flow.

**Figure 1:**
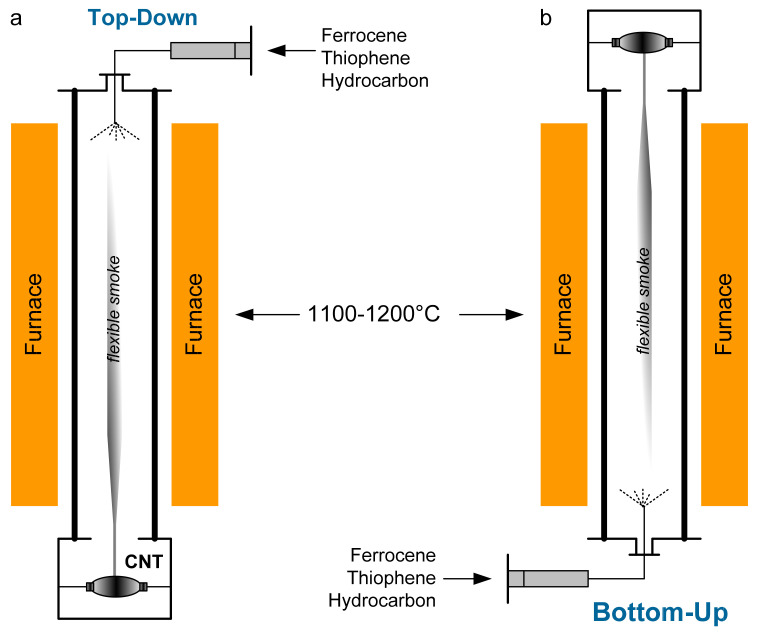
Schematic drawing of a laboratory reactor block for the synthesis of nanotubes with in situ winding: (**a**) top-down “Cambridge Process” [14]; and (**b**) bottom-up “Moscow Process” [16].

Synthesis of “flexible smoke” of nanotubes in a gas-carrier flow is usually carried out in a quartz reactor at a temperature of 1100–1200 °C. A three-component reaction mixture of a carbon precursor (ethanol, butanol, acetone, etc.), thiophene and a catalyst is fed into the reactor from top-to-bottom (Cambridge Process) [14] or bottom-to-top (Moscow Process) [16] with a gas-carrier flow. The catalyst that was suggested by Windle et al. in [14] is Fe nanoparticles, which are generated by thermal decomposition of ferrocene (C_2_H_5_)_2_Fe. The scheme of the laboratory reactor block with a quartz reactor for the aerosol catalytic synthesis of carbon nanotubes with winding top-down and bottom-up is shown in Figure 1. In this case, the method of supplying the reaction mixture (top-down or bottom-up) does not affect the formation of a nanotube in the form of a stocking or a sprout. The “flexible smoke” is pulled out of the reactor using a special grip from the bottom or top (Figure 1). A very fine sprout/stocking is formed and wound onto a spool. At the same time, the problem of synthesizing high-quality carbon nanotubes without residual catalyst impurities and non-CNT remains urgent. There are also no systematic data in the literature on the use of other metallocenes or their mixtures, although this would be a good way to improve process control.

Ferrocene is the most used catalyst. There are many publications on the use of ferrocene for the synthesis of carbon nanotubes by the CVD method and the study of their properties [14,18,19,20,21,22,23,24,25,26]. There are publications where cobaltocene [27] and nickelocene [28,29] or their combinations with ferrocene are used as catalysts, but they are few [30,31,32,33]. Some data from such publications are presented in Table 1.

In article [23], the formation of an aerogel of nanotubes using nickelocene as a catalyst was not observed. Small clusters of nanotubes formed only after the addition of sulfur. The authors note the effect of sulfur on the catalyst and the formation of a continuous aerogel. Continuous formation of a nanotube aerogel stocking was observed using ferrocene or cobaltocene. Nanotubes obtained using cobaltocene had the same characteristics as nanotubes obtained from ferrocene.

The publication [30] describes the pyrolysis of benzene in the presence of monometallocene vapors—Fe, Co and Ni, leading to the formation of nucleation centers for the growth of nanotubes. Along the way, onion-like structures filled with metal are formed. It is noted that at high flow rates of the gas mixture and low ferrocene content, the yield of nanotubes increases, while the wall thickness and diameter of nanotubes significantly decrease. However, pyrolysis of benzene in the absence of any metallocene at a temperature of 1140 °C leads to the formation of monodisperse carbon nanospheres with a diameter of appr. 200–500 nm.

In article [31], a mixed solution of metallocenes in benzene was sprayed into a reactor. The product precipitated out as a black “flaky” powder. The product yield increased significantly when benzene was sprayed with a mixture of Fe:Ni metallocenes. Especially many nanotubes were synthesized when using a mixture of metallocenes Fe:Ni = 65:35. This Fe:Ni ratio significantly improved the product purity and the yield of aligned CNT structures. It is noted that when using an aerosol with an Fe:Ni catalyst, the diameter of the nanotube decreases, and along the length of the nanotube it is filled with a metal component. However, with both ferrocene and an Fe:Ni mixture, nanotubes with a bimodal diameter distribution are reported (Table 1).

Varying the compositions of mixtures of Fe:Ni catalysts [32] with carbon black (CB) carbon led to the formation of nanostructures of different morphology, but with a higher degree of graphitization than when using single metallocene only. Small changes in the composition of the Fe:Ni catalyst mixture affected the solubility of carbon in the catalytic system. This influenced the growth rate of nanotubes, yield and degree of crystallinity. An increase in the yield of nanotubes and the degree of graphitization were observed when the ferrocene content was high. The authors note that nanoscale structures with a high degree of crystallinity were obtained at mass ratios CB:ferrocene:nickelocene = 1:2:2.

The analysis of the publications cited in Table 1 shows that the conditions for the synthesis of nanotubes (temperature, carbon precursor and other parameters) are very different and, accordingly, the resulting product is also different. There have been no systematic studies in literature yet. The publications on metallocene catalysts for the production of CNTs over the past two decades do not give a complete pattern.

It is worth noticing that, application-wise, the use of mono-, bi-, and tri-metallocenes of the group VIII (Fe, Co, Ni) will be a good way to improve the control of the process of the nanotube production and provide prospects for emerging nanotube applications such as solar cells, electromagnetic shielding or lithium-sulfur batteries.

The purpose of this work is to study how the use of single or mixed metallocenes of group VIII (Fe, Co, Ni) influences the parameters of aerosol CNT synthesis realized by the method realized in our earlier work [17] for ferrocene only and the properties of CNT produced.

## 2. Materials and Methods

**Figure 2:**
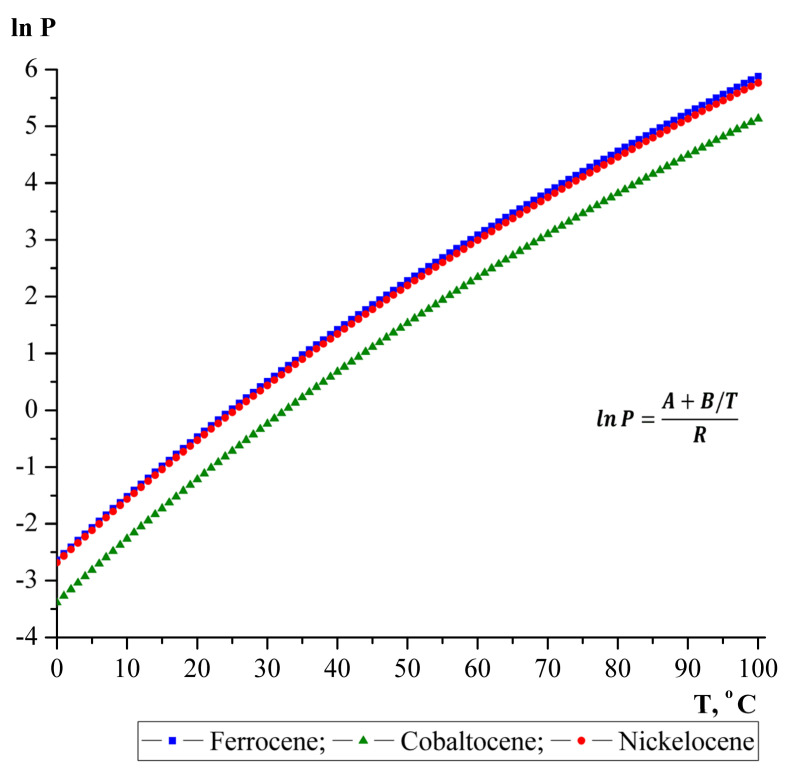
Temperature dependence of the logarithm of vapor pressure (metallocenes Fe, Co and Ni).

In this work, ferrocene, cobaltocene, and nickelocene were used as catalyst precursors. These metallocenes have similar melting points and relatively low sublimation temperatures (150–300 °C). Vapor pressure and their temperature dependencies seem important for application in the aerosol synthesis. Calculations were performed based on the data available in the literature [34]. The results of the calculated data on the temperature dependence of the vapor pressure of the Fe, Co and Ni metallocenes are displayed graphically in Figure 2. The saturated vapor pressure of the group VIII metallocenes was calculated according to the Antoine equation in the temperature range 0–100 °C:

R × lnP = A + B/T(1)

P = exp (A + B/T)/R(2)

where: R—gas constant, A and B—empirical constants for each specific catalyst [34]: for ferrocene—A = 242.09 ± 1.23 J K^−1^ mol^−1^; B = −72073 ± 362 J mol^−1^; for cobaltocene—A = 235.95 ± 0.36 J K^−1^ mol^−1^; B = −72095 ± 111 J mol^−1^; for nickelocene—A = 239.51 ± 2.14 J K^−1^ mol^−1^; B = −71468 ± 630 J mol^−1^.

The linear dependence of the logarithm of the vapor pressure of the Fe, Co and Ni metallocenes on temperature indicates that with increasing temperature, the vapor pressure increases due to an increase in the concentration of metallocene molecules. However, the cobaltocene has a slightly lower vapor pressure than nickelocene and ferrocene at the same temperature. Nevertheless, we can say that the processes of evaporation and nucleation of nanotubes on active cobaltocene and nickelocene nanoparticles along the reactor axis will be the same as on ferrocene nanoparticles. Accordingly, the characteristics of the synthesized nanotubes will differ slightly. In order to confirm these assumptions, a series of experimental works was carried out. For the synthesis of nanotubes, we used both single metallocenes, Fe, Co and Ni, and their various combinations.

**Figure 3:**
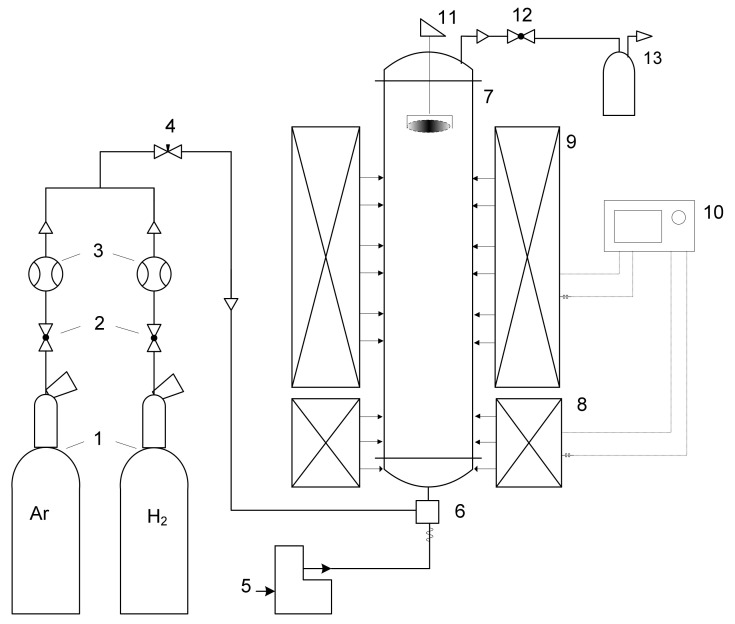
Schematic representation of the laboratory setup: 1—He, H_2_ cylinders; 2, 12—valve; 3—mass flow controller; 4—fine regulation valve; 5—liquid phase feeder; 6—mixer evaporator; 7—quartz tube reactor; 8—preheater; 9—furnace; 10—temperature regulator; 11—rotating harvester; 13—filter system.

The CNT synthesis experiments were carried out in a laboratory setup in a vertical flow quartz reactor (Figure 3). CNTs were synthesized on the surface of Fe, Co and Ni catalytic particles suspended in a carrier gas flow. The reaction vapor–gas mixture of ethanol, thiophene and catalyst in a stream of hydrogen entered the synthesis zone of the reactor from the bottom entrance of the reactor. The ratios of the starting reagents corresponded to the composition of the mixture reported elsewhere [17]. The concentration of the catalyst for the continuous growth of the CNT aerogel was kept 1 wt%. CNTs were grown at a reaction temperature of 1150 °C. The synthesized CNT product was collected in the cold zone at the top part of the reactor.

Table 2 shows the ratios of the used precursors of metallocene catalysts Fe, Co and Ni and their various combinations for aerosol synthesis. A brief description of the resulting carbon product is given.

The structural properties of carbon nanotubes were studied by high-resolution transmission and scanning electron microscopy with the use of JEM-2010 (TEM) equipped by Energy-dispersive X-ray spectroscopy (EDS) and electron energy loss spectroscopy (EELS) attachments; and JEOL JSM-7600F (SEM). Electrical resistivity was measured by the four-probe method, using Keithley 4200 SCS. The samples for electrical resistivity measurements were prepared by rolling the nanotube samples into flat strips with length of 50 to 80 mm, width of 10 mm and thickness of 18 to 28 µm. The content of the residual catalyst (metal particles residing inside and/or outside of CNT) was determined by thermogravimetric analysis (TGA). The Raman spectra were recorded with a Renishaw inVia confocal Raman microscope with 532 nm laser excitation.

## 3. Results and Discussion

The results shown in Table 2 are remarkable by the fact that almost all the CNT were received mainly in the form of an aerogel “stocking”. A “stocking” aka “sprout” is a semi-transparent hollow cylinder, which appears in the bottom section of the reactor and grows up through the reactor reaching the top and then getting deposited in the receiving chamber as described in detail elsewhere [16]. Only in the case of using 100% nickelocene and the ratio of mixtures of metallocenes Fe:Ni and Fe:Co equal to 25:75 (i.e., in case of great excess of nickelocene or cobaltocene) entangled threads (no appearance of a “stocking”, just short entangled thread-like collections of carbon material flying through the reactor and getting deposited in a receiving chamber) are obtained. In other respects, continuous growth of the nanotube “stocking/sprout” was observed.

**Figure 4:**
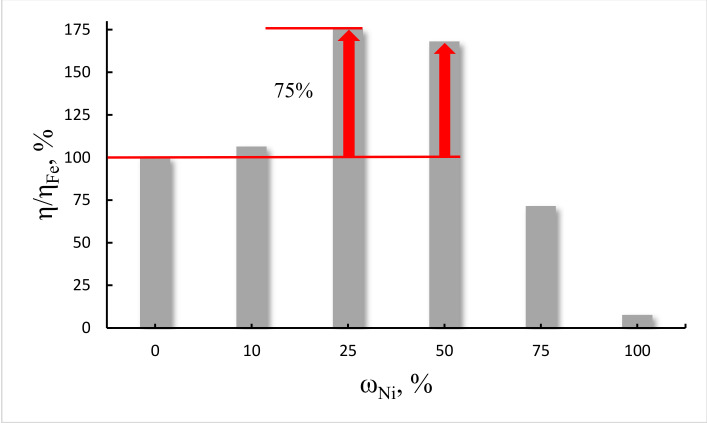
Relative yield η/η_Fe_ of the nanotubes at various compositions of the catalyst. Extremums are observed at the ratio Fe:Ni = 50:50 and 75:25. Bimetallic Fe-Ni catalyst pushes the relative yield up to 175% (with the Fe catalyst as a 100% reference).

A strong dependence of the yield of nanotubes on the catalyst composition was revealed. So, the Figure 4 shows how the product yield changes depending on the nickelocene content in the Fe-Ni bimetallic catalyst. The histogram shows that the nickelocene content of 25–50 wt% in the Fe-Ni bimetallic catalyst significantly increases the nanotube yield up to 75%. However, if the content of nickelocene in the Fe-Ni catalyst is higher than 50% or lower than 25%, then the yield of nanotubes decreases. Such an extreme effect on the yield of CNTs may be due to the lowest energy of solubility of carbon in active particles of Fe and Ni at metallocene ratios Fe:Ni = 50:50 and Fe:Ni = 75:25. The high iron content in the Fe:Ni bimetallic catalyst shows the higher activity of this component for the growth of nanotubes compared to other mass ratios. This indicates that the solubility of carbon in the Fe nanoparticle is higher than in the Ni nanoparticle. In [35] it was noted that the solubility of carbon determines the number of layers. Although, in the resulting carbon product, there are both thin-walled and multi-walled nanotubes.

So, Fe Ni bimetallocene catalysts with a high iron content show a higher activity of nanotube growth than when the nickelocene is greater than 50 wt% or less than 25 wt%. A similar extreme character of the dependence of the yield of nanotubes on the catalyst composition was also found for a bimetallocene Fe:Co catalyst with an extremum at Fe:Co = 50:50 and Fe:Co = 75:25 ratios. Fe:Co metallocene catalysts with a higher iron content also exhibit higher catalytic activity in the process of nanotube growth.

It can be assumed that the solubility of carbon in the Fe nanoparticle is higher than in Co and Ni and, therefore, the possibility of the formation of aerogel nanotubes is greater in a mixture with a high content of Fe nanoparticles. The authors of [36] note that bimetallic catalysts give higher growth rates of nanotubes and lower synthesis temperatures than monometallic catalysts. However, in this article, a mixture of Fe-Ni catalysts is the most favorable for the growth of carbon structures of nanofibers and nanotubes at a low synthesis temperature. In our work, we synthesized nanotubes at a higher temperature, and the effect of carbon diffusion also proceeds faster for bimetallic catalysts Fe-Co and Fe-Ni than for monometallocenes Co or Ni.

Summarizing the above, we can conclude that carbon atoms diffuse faster in bimetallic Fe-Co and Fe-Ni catalysts with a high iron content than in monometallic catalysts Co and Ni. Here it is necessary to note the manifestation of the synergistic effect of the bimetallic catalyst. There are a lot of publications on the synergistic effect that is observed when two or more metals are combined in a catalyst [29,33,37,38,39]. The interdiffusion of two or three active nanoparticles in comparison with one nanoparticle significantly increases not only the growth rate of nanotubes, but also improves the uniformity and quality of the product, gives a narrower nanoparticle size distribution, etc.

**Figure 5:**
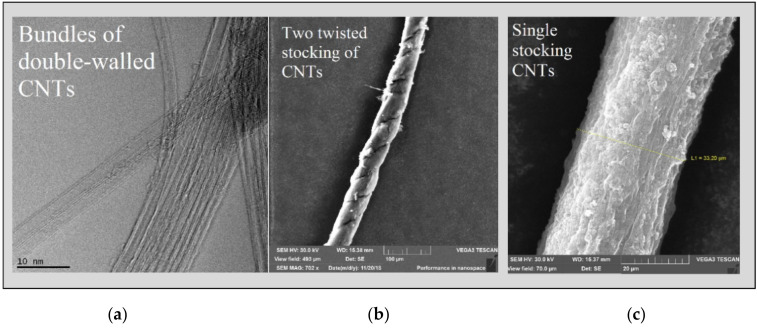
Electron microscopy images of the nanotubes produced with ferrocene as a catalyst precursor: (**a**) TEM of double-walled nanotube bundles; (**b**) Low resolution SEM of a thread twisted from two nanotube sprouts stocking; (**c**) a thread produced from a single nanotube sprout.

The mechanism of the growth of nanotubes on the surface of an active metal occurs due to the diffusion of carbon through a metal particle, as well as the segregation of carbon in the form of ordered graphene layers [40]. In our experiments, the continuous segregation of carbon in the form of graphene layers leads to the formation of a continuous pack of nanotubes in the form of a “stocking/sprout” as described above in this paper. Studies of such a CNT stocking made of ferrocene using an electron microscope showed that they mainly consist of bundles of long double-walled nanotubes (up to 70%) with a diameter distribution of 1.5–3.5 nm and a very high length (Figure 5a). The diameter of a CNT sprout consisting of bundles of nanotubes is usually 20–35 μm (Figure 5c). Two of these single sprouts can be twisted into a thread (Figure 5b). According to EDX and TGA data, the iron content in the initial crude CNTs is 2–10 wt%., while in the purified ones 0.2–2 wt%.

**Figure 6:**
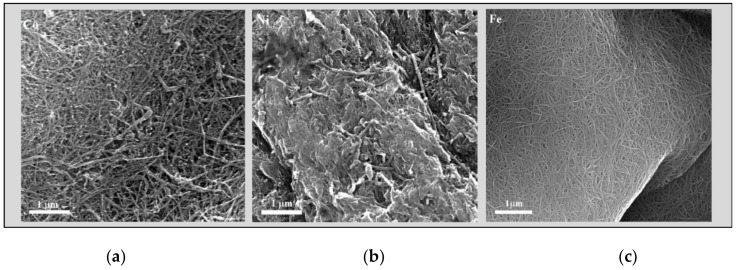
SEM images of the carbon nanotubes synthesized over different metallocenes: (**a**) cobaltocene; (**b**) nickelocene; (**c**) ferrocene.

It was noted above that the presence of nanoparticles with high content of Ni and Co in the reaction medium affects the formation of the final carbon product (Table 1). When using nickelocene or increasing the total mass of nickelocene or cobaltocene in the bimetallic catalyst (up to 75% or more), the formation of a continuous aerogel of the CNT stocking was not observed; carbon nanotubes were formed in the form of entangled threads. High-resolution SEM studies of nanotubes have shown that, depending on the catalyst used, the morphology of nanotubes is very different. The SEM images are shown in Figure 6. It is clearly seen how the morphology of nanotube arrays changes. It should be noted that in order to obtain a clear image of the nanotube arrays, the samples under study were preliminarily heat treated and chemically purified by 37% HCl from the residual catalyst [41].

**Figure 7:**
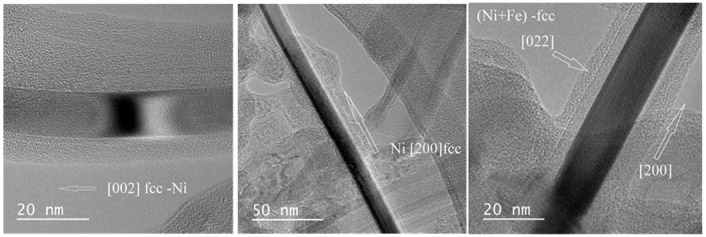
TEM images of single crystal metal (Ni or Ni+Fe) nanokernels grown inside nanotubes with [200] orientation along the nanotube axis. The left and central images show Ni monocrystals in different directions; the right image demonstrates Ni+Fe crystal.

**Figure 8:**
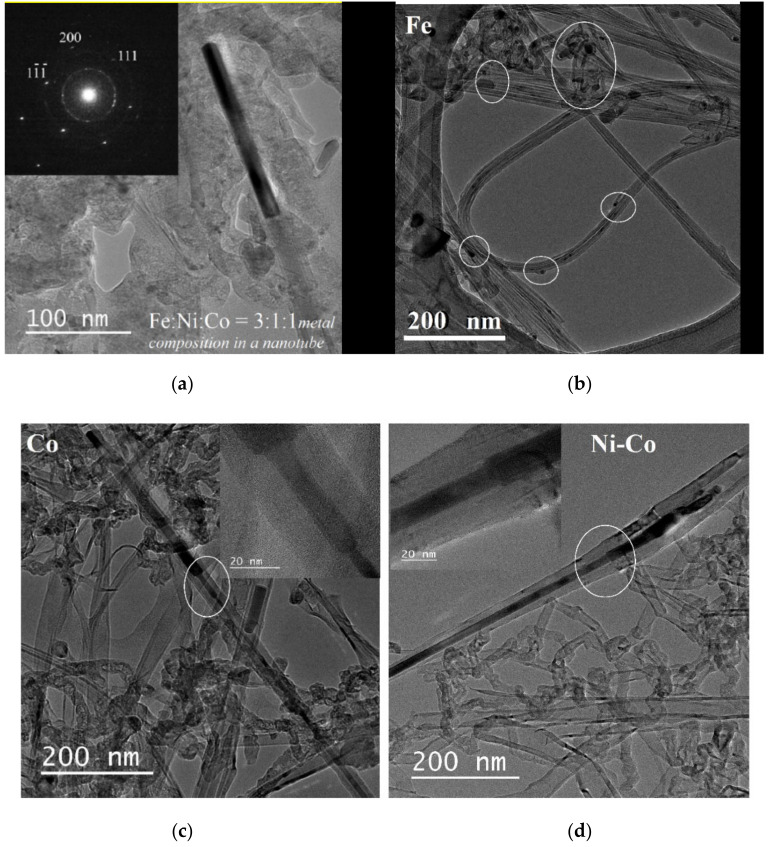
TEM images of the nanotubes with internal metal nanokernels: (**a**) from a mixture of metallocenes Fe-Co-Ni alloy in the growth direction [200] (diffraction pattern inserted); (**b**) from ferrocene with iron encapsulated in a carbon shell; (**c**) from cobaltocene-segmented nanotubes in the bulk and a Co-filled nanotube with a length of more than 1000 nm; (**d**) from a mixture of Ni-Co metallocenes with a length of more than 1000 nm.

Studies of CNT samples by TEM (HRTEM—High-resolution transmission electron microscopy) showed the formation of metallic inclusions in the nanotubes (Figure 7 and Figure 8). In the inner channels of carbon nanotubes, “nanowire” or “nanorod” particles are grown from nickel, cobalt and alloys of these metals with iron forming what we can call a kernel. The electron diffraction study showed that the kernels comprise of metal phases and consist of single crystals aligned along the nanotube axes. Figure 7 shows nanotubes containing Ni and Ni-Fe kernels. All three shown kernels manifest an fcc structure and are oriented in the growth direction [200].

An interesting result was obtained for a nanotube containing an Fe-Ni-Co metal alloy (Figure 8a). Combination of three active catalyst metals leads to the same orientation of the fcc nanokernel in the nanotube in the growth direction [200]. It also shows the diffraction pattern of this unusual object made of an Fe-Ni-Co metal alloy. EELS confirmed the presence of an alloy of all three metals in the nanokernel, where ratio Fe:Ni:Co = 3:1:1. Despite the fact that the ratio of the initial mixture of three metallocenes was 1:1:1 (Table 2), the iron content in the alloy always prevails. This effect is also typical for bimetallocene catalysts Fe:Ni and Fe:Co.

In nanotube samples received using cobaltocene (Figure 8c) and a mixture of cobaltocene with nickelocene (Figure 8d), the formation of nanokernels with a stepped protrusion is observed, the cylindrical structure of the nanotube is deformed. Perhaps this effect is induced by a particle of cobalt. Nevertheless, active catalyst particles continue to be sucked into the growing nanotube, which leads to the formation of metal nanokernels with Co and Ni-Co alloy with a length of more than 1000 nm. The diffraction pattern of the metal fillers showed the presence of pure Co single crystals and the Ni-Co alloy, respectively.

A different picture is observed for CNTs obtained from ferrocene (Figure 8b). It can be seen that the catalyst particles are not located inside nanotubes but are separately encapsulated in a carbon shell. EELS confirmed the presence of iron particles in the out-of-CNT shells. At the same time, no catalytic metal particles were observed inside the nanotubes. Perhaps, as noted above, this is due to the higher solubility of carbon in the Fe nanoparticle than in Co and Ni.

**Figure 9:**
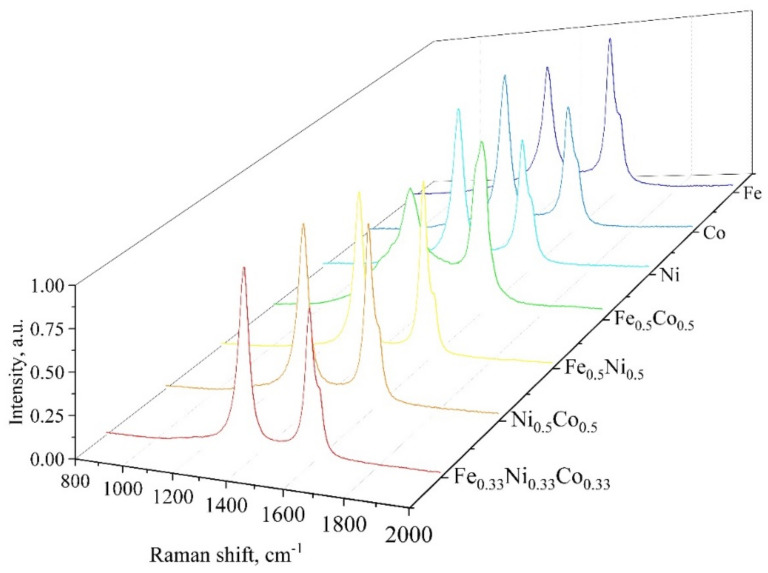
Raman spectra of the carbon nanotubes produced over different metallocene-derived catalysts.

The electron microscopy data suggest that all the catalysts under investigation produced relatively thin multiwalled nanotubes accompanied with non-nanotube carbon. There is a tendency of formation of nanotubes with poorer structure (curved, bamboo-type, etc.) over bimetallic catalysts. These differences may show up in Raman spectra of the produced nanotubes. However, the Raman spectra do not manifest any great difference as can be seen from Figure 9. Indeed, all the spectra are characterized by prominent D- and G- peaks showing the presence of nanoscale graphene structures, which is typical for thin-walled and multiwalled carbon nanotubes. No radial breathing mode observed, which is presumably due to no single-walled nanotubes as well as to the presence of the intra-tube metal kernels. The nanotubes produced over ferrocene are the only ones that are characterized by a G-peak dominating D-peak, which is consistent with electron microscopy data. The nanotubes produced over bimetallic ferro-cobaltocene manifest very broad D-peak, which is presumably due to a variety of non-nanotube carbons present and poorer structure of the nanotubes themselves.

**Figure 10:**
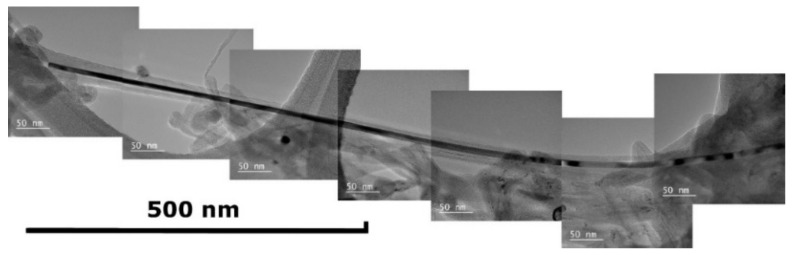
TEM image of a carbon nanotube with extra-long monocrystal Ni kernel inside.

The capillary effect of filling a growing carbon nanotube was discovered using both a single metallocene and bi- and trimetallocene mixtures. Filling a nanotube with a catalytic particle leads to the formation of unusually long strait kernels, which grow longer than 1000 nm and even 1500 nm (Figure 10). The degree of filling a nanotube with a metal particle depends on the type or combination of catalysts used. It is clearly seen in Figure 10 where the nickel nanokernel starts its growth while it is impossible to observe where it breaks off.

Measurements of the electrical resistance of CNT samples obtained on metallocenes Fe, Co, Ni and their combinations in various ratios were carried out. The results of measurements showed that the resistivity of nanotubes can vary over a wide range from 1.0 × 10^−3^ to 1.1 × 10^−5^ Ω∙m. The electrical characteristics of the nanotube samples are presented in Table 3. Such strong difference in electrical resistivity can be explained both by peculiarities in CNT morphology and possible contribution by monocrystal nanokernels inside the nanotubes. The nature of the nanokernel influence on physical properties is yet to be researched.

Measurements of the electrical resistance of CNT samples obtained on metallocenes Fe, Co, Ni and their combinations in various ratios were carried out. The results of measurements showed that the resistivity of nanotubes can vary over a wide range from 1.0 × 10^−3^ to 1.1 × 10^−5^ Ω∙m. The electrical characteristics of the nanotube samples are presented in Table 3. So strong a difference in electrical resistivity can be explained both by peculiarities in CNT morphology and possible contribution by monocrystal nanokernels inside the nanotubes. The nature of the nanokernel influence on physical properties is yet to be researched.

## 4. Conclusions

Aerosol carbon nanotubes were obtained using metallocene catalysts Fe, Co and Ni in various combinations. The influence of the catalyst composition variation on the structure, yield, degree of filling and purity of the resulting carbon nanotubes was revealed.

A number of unexpected cooperative effects are reported when mixed metallocenes are used in the synthesis of CNTs which are not observed in case of individual metallocenes, such as the commonly used ferrocene catalyst Fe(C_5_H_5_)_2_. The formation of very long (up to several µm) straight monocrystal metal kernels inside the carbon nanotubes was the most interesting effect. The use of trimetal catalysts (Fe_1-x-y_Co_x_Ni_y_)(C_5_H_5_)_2_ resulted in the sharp increase in the yield of carbon nanotubes. It was found that interdiffusion of two or three active nanoparticles in comparison with one nanoparticle significantly increases not only the growth rate of nanotubes, but also improves the uniformity and quality of the product, gives a narrower nanoparticle size distribution, etc. As for the composition of the inside-CNT nanokernels, the concentration of Fe was always higher than in the feeding mixture of metallocenes. The electrical conductivity of produced nanotubes is determined by the nature of the catalyst. The variation of individual metals in the Ni-Co-Fe leads to a drop of the electrical resistivity of nanotube samples by the order of magnitude, i.e., from 1.0 × 10^−3^ to 1.1 × 10^−5^ Ω∙m. A controlled change in the electrophysical properties of the nanotubes can make it possible to expand their use as fillers in composites, photothermal and tunable magnetic nanomaterials with pre-designed electrical conductivity and other electromagnetic properties [42,43].

## Figures and Tables

**Table 1:** Experimental conditions and properties of carbon nanotubes obtained on metallocene catalysts Fe, Co and Ni by aerosol synthesis.

#	Precursor	Synthesis Conditions	Products	References
1	Ferrocene	Methane, Thiophene, 1290 °C, H_2_, plasma spark generator	The outer diameter of bundled CNT is 6–40 nm; length over 100 μm	[23]
	Cobaltocene			
	Nickelocene		Clusters of curled short CNTs with varying outer diameter	
2	Ferrocene	Benzene, 900 °C, Ar:H_2_ = 3:1;17:3 Flow rate 50, 1000 sccm, quartz boat	MWCNTs, outer diameter 40–90 nm and metal-filled onion-like structures	[30]
	Cobaltocene			
	Nickelocene			
3	Ferrocene	Benzole, 800–950 °C, Ar, “atomised”	Thick CNTs, outer diameter 60–120 nm and 90–200 nm. Thin CNTs, outer diameter: 10–40 nm and 10–70 nm (more than 4–10 walls).	[31]
	Ferrocene: Nickelocene (25:75), (65:35)			
4	CB *:ferrocene:nickelocene (9.1:45:45) (9.1:91:0) (12.5:62.5:25) (12.5:25:62.5) (20:40:40)	Toluene, 1000 °C, N_2_, alumina boat	MWCNT and bulbous structures. Outer diameter: 20–150 nm 50 nm 10–100 nm 10–50 nm 10–30 nm	[32]

* Carbon black.

**Table 2:** The metallocene catalysts used in this work.

No	Catalyst Precursor *	Metal	Ratio	Product
1	Ferrocene	Fe	100	“stocking”
2	Nickelocene	Ni	100	threads
3	Cobaltocene	Co	100	“stocking”
4	Ferrocene/Nickelocene	Fe:Ni	25:75	threads
5	Ferrocene/Nickelocene	Fe:Ni	50:50	“stocking”
6	Ferrocene/Nickelocene	Fe:Ni	75:25	“stocking”
7	Ferrocene/Nickelocene	Fe:Ni	90:10	“stocking”
8	Ferrocene/Cobaltocene	Fe:Co	25:75	threads
9	Ferrocene/Cobaltocene	Fe:Co	50:50	“stocking”
10	Ferrocene/Cobaltocene	Fe:Co	75:25	“stocking”
11	Nickelocene/Cobaltocene	Ni:Co	50:50	“stocking”
12	Ferrocene/Nickelocene/Cobaltocene	Fe:Ni:Co	33:33:33	“stocking”

* The total concentration of metallocenes in the reaction mixture was 1 wt%.

**Table 3:** Electrophysical and structural characterization of the nanotubes synthesized with different catalysts.

No	Active Metal in Catalyst	Ratio	Catalyst Residual Content in CNT, wt% *	Electrical Properties		Description of Nanotubes
				Resistivity, Ω∙m	Conductivity, S/m	
1	Fe	100	5.5	3.3 × 10^−4^	3.0 × 10^3^	straight
2	Ni	100	6.3	6.7 × 10^−3^	1.5 × 10^2^	straight
3	Co	100	7.4	8.3 × 10^−4^	1.2 × 10^3^	segmented
4	Fe:Ni	25:75	2.4	8.3 × 10^−3^	1.2 × 10^2^	straight
5	Fe:Ni	50:50	3.7	4.3 × 10^−4^	2.3 × 10^3^	straight
6	Fe:Ni	75:25	5.6	1.9 × 10^−4^	5.4 × 10^3^	straight
7	Fe:Ni	90:10	2.5	1.2 × 10^−4^	8.2 × 10^3^	straight
8	Fe:Co	25:75	6.1	1.5 × 10^−3^	6.5 × 10^2^	straight, segmented
9	Fe:Co	50:50	6.3	5.9 × 10^−4^	1.7 × 10^3^	straight, segmented
10	Fe:Co	75:25	9.9	3.7 × 10^−4^	2.7 × 10^3^	straight, segmented
11	Ni:Co	50:50	1.8	1.8 × 10^−3^	5.5 × 10^2^	straight, segmented
12	Fe:Ni:Co	33:33:33	3.8	1.0 × 10^−3^	1.0 × 10^3^	straight, segmented
13	Fe	100	0.2	1.1 × 10^−5^	9.4 × 10^4^	straight, purified

* measurement error 2.0 wt%.
